# FAAST: Flow-space Assisted Alignment Search Tool

**DOI:** 10.1186/1471-2105-12-293

**Published:** 2011-07-19

**Authors:** Fredrik Lysholm, Björn Andersson, Bengt Persson

**Affiliations:** 1 IFM Bioinformatics and SeRC (Swedish e-Science Research Centre), Linköping University, S-581 83 Linköping, Sweden; 2 Department of Cell and Molecular Biology, Science for Life Laboratory, Karolinska Institutet, S-171 77 Stockholm, Sweden

## Abstract

**Background:**

High throughput pyrosequencing (454 sequencing) is the major sequencing platform for producing long read high throughput data. While most other sequencing techniques produce reading errors mainly comparable with substitutions, pyrosequencing produce errors mainly comparable with gaps. These errors are less efficiently detected by most conventional alignment programs and may produce inaccurate alignments.

**Results:**

We suggest a novel algorithm for calculating the optimal local alignment which utilises flowpeak information in order to improve alignment accuracy. Flowpeak information can be retained from a 454 sequencing run through interpretation of the binary SFF-file format. This novel algorithm has been implemented in a program named FAAST (Flow-space Assisted Alignment Search Tool).

**Conclusions:**

We present and discuss the results of simulations that show that FAAST, through the use of the novel algorithm, can gain several percentage points of accuracy compared to Smith-Waterman-Gotoh alignments, depending on the 454 data quality. Furthermore, through an efficient multi-thread aware implementation, FAAST is able to perform these high quality alignments at high speed.

The tool is available at http://www.ifm.liu.se/bioinfo/

## Background

The nature of DNA sequencing has taken a dramatic turn in the last few years, most notably improved through the development and broad use of 2nd generation sequencing methods. The first 2nd generation sequencing method was 454 sequencing, introduced in 2005 with the GS20 sequencing machine which produced 20 million base-pairs (Mbp) per run [1]. 454 sequencing has since been improved steadily both regarding quality and throughput, and the GS FLX Titanium, introduced in 2008, produces 500 Mbp per run, as reads of approximately 350 bp [2]. Although, since 2005, other 2nd generation sequencing methods have emerged, 454 still produces the longest reads and is one of the most widely used platforms. The long reads produced by 454 sequencing makes the method especially attractive for metagenomic sequencing, where the sample is highly complex and overlapping reads are more rare. The enormous technology improvements represented by novel sequencing technologies do not only enable many new studies but also poses great challenges in terms of processing the sequence data. The major underlying technology for data processing is sequence alignment, which plays a key part in all steps from sequence assembly to annotation.

In 1970, the global sequence alignment was proposed and a computational method for solving it [3]. The algorithm utilised the fact that the problem can be solved through solving a number of sub-problems, dynamic programming, which greatly reduced the number of pathways to explore. A decade later, through a modified dynamic programming algorithm, Smith and Waterman defined the local alignment and a method for solving it [4]. Yet another year later Gotoh added non-linear gap penalties to the algorithm [5]. In terms of accuracy dynamic programming methods are still the preeminent methods for solving the two problems and later methods such as FASTA [6,7] and BLAST [8,9] use the same underlying technology to calculate alignments.

**Figure 1:**
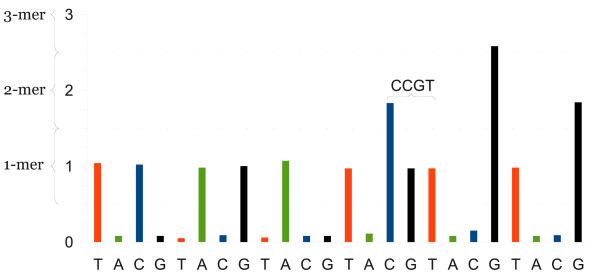
**Example of a flowgram**. A 'flowgram' where 'flowpeak' values provided on the y-axis are proportional to the number of nucleotides found at each position.

The traits of 454 data are different from those of other sequencing techniques, which occasionally cause problems for computer analysis software. To minimise the effect of sequencing error and maximise the efficiency of 454 sequencing, it is crucial to consider the particular characteristics of 454 data while computing alignments. 454 sequencing is a pyrosequencing method, where DNA fragments are associated with synthetic beads in picolitre sized reactor wells and sequenced in parallel. Nucleotide reagents for detection of thymine (T), adenine (A), cytosine (C) and guanine (G) are repeatedly cycled over the DNA template fragments while elongating the complementary strand [1]. The intensity of each reaction is recorded, as a so-called flowpeak, by a CCD camera for each well where the intensity is proportional to the length of the homopolymer at that position, see Figure 1. A flowed base in which the complementary strand is not elongated is denoted as a *negative flow *and consequently flows in which it is elongated are denoted as *positive flows*, i.e. flow peaks < 0.5 and ≥ 0.5, respectively [1]. For example, the flows A(0.11), C(1.83), G(0.97) and T(0.97) would be one *negative *and three *positive flows *most likely produced by a "CCGT" *4-mer*. Because the homopolymer length is estimated from the flowpeak value, homopolymer indels (insertion or deletion) is the most common type of reading error. To produce a substitution reading error, an undercall must be followed by an overcall, or vice versa [1]. At the same time, many scattered indels are normally more rare than dispersed substitutions, and therefore, in standard sequence alignments, more heavily punished [10]. As a consequence, current alignment search programs are more optimised towards detecting alignments with occasional substitutions rather than many small gaps.

Recently, to improve the alignment quality using 454 reads, an attempt was made to utilise the flowgram information through probabilistic flowgram matching [11]. The downside of using flowgram matching, i.e. direct matching of flowgrams, is that an SNP will either shift the flowgram one cycle or be matched as two insertions, resulting in an insignificant hit or a hit of low significance, respectively. As SNPs, if not already a factor, also occur as PCR artefacts in sequencing, direct flowgram matching can only be used in conjunction with sequence alignment to improve the accuracy in the cases where homopolymer ambiguity affects the results. Another tool named PanGEA [12] employs a dynamic gap penalty for alignments where the gap penalties are decreased with an increasing homopolymer. The downside of PanGEA is that it does not consider the pyrosequencing flowpeak values and also uses a linear gap-extension penalty for homopolymer correcting gaps.

Through combining the ability to correct for homopolymer reading errors with sequence alignments, more accurate alignments of 454 data can be achieved. To address these problems, we suggest the use of flow-space assisted Smith-Waterman-Gotoh alignments, i.e. giving the local alignment algorithm the ability to correct for likely sequencing errors while computing the alignment. We implemented the flow-space assisted Smith-Waterman-Gotoh alignment algorithm in a C++ tool named FAAST (Flow-space Assisted Alignment Search Tool) and performed alignments using both regular Smith-Waterman-Gotoh alignments and FAAST.

## Results

### Evaluation of the effect of flow-space assisted local alignment

By introducing the possibility to perform flow-peak correction, the 'degrees of freedom' for the maximum likelihood estimate increases, potentially producing untrue alignments. For example, if any flow-peak correction was allowed without penalty, any flowgram could match any sequence identically. Therefore, an extensive study of the effect of the flow-space assisted local alignment is needed. The model used for the Smith-Waterman-Gotoh alignment is match/mismatch score = 2/-3 and gap open/extended penalty 5/2, and the additional parameter (see Methods) for the flow-space assisted local alignment is program default (*k *= 0.25).

Three targets of 25, 50 and 100 nucleotides were randomly picked from the ethidium bromide resistance determinant of *Staphylococcus epidermidis *(NC_003969), see additional files. For each of these an additional 100 decoy sequences were generated, resulting in a database of 101 nucleotide sequences. The decoys were generated through introducing random SNPs corresponding to 92% identity. This small sequence set would represent the homologs found in an everyday database search.

Finally, query sequences were generated from the target sequence and the algorithms were assessed on their ability to recover the target sequence as the highest scoring alignment, thus find the 'correct' homology in a set of similar decoys. The query sequences were generated ranging from 100% down to 72% nucleotide identity (through introducing random SNPs) using no 454 data simulation (Plain) as well as using Flowsim [13] to simulate 454 data. 454 data was generated through Flowsim with the generation settings (-G) set to 'Titanium' and 'GS20', as well as a 'high noise' model. The 'high noise'-model constituted a LogNormal(-2.5, 0.2) distribution for *negative flows *and a Normal(*n*, 0.15*n) distribution for *positive flows *of length *n*.

**Figure 2:**
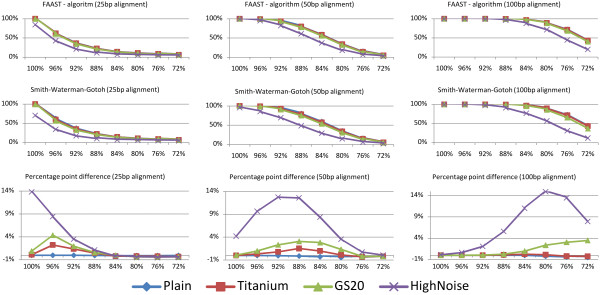
**Evaluation of the effects of flow-space assisted local alignment**. Showing the rate of recovered target sequence (per cent on y-axis) at dropping query-target identity (x-axis) among 100 decoys generated at 92% identity to each target sequence. The test was performed for three different targets of 25, 50 and 100 bp, using FAAST and Smith-Waterman-Gotoh alignment.

The results were evaluated using FAAST with the homopolymer penalty regulating parameter at *k *= 0 (Smith-Waterman-Gotoh algorithm) and *k *= 0.25 (FAAST algorithm) and to provide stable means each sub-test ran 10,000 times, see Figure 2. In general, as expected, the accuracy drops as the identity between the generated query sequence and the target sequence decreases. When the decoy sequences are more similar to the target than the generated query sequence it is hard for both algorithms to find the target. Also, with an increasing alignment length (i.e. 100 compared to 50 compared to 25), the accuracy in terms of per cent 'correct' homologs identified increases as more non-SNPs positions still match. For alignments of query sequences not passed through Flowsim (non-454-like data), the Smith-Waterman-Gotoh algorithm slightly outperforms FAAST. However, for Titanium up to 'high noise' 454 data the alignment results are generally improved by flow-peak correction.

**Figure 3:**
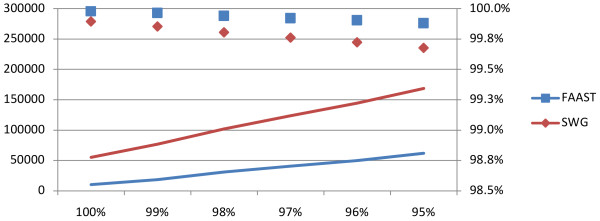
**Mapping evaluation of flow-space assisted local alignment**. Showing the number of incorrectly aligned nucleotides (lines, left y-axis) as well as the per cent correctly aligned nucleotides (diamonds/squares). The test was performed through simulating 100,000 Titanium reads, at 100% down to 95% identity (x-axis) from *Salmonella enterica subsp. enterica serovar Typhi str. CT18*. The reads were finally aligned back against the genome and number of correctly aligned nucleotides was assessed.

To further test the effect of the FAAST algorithm we evaluated the number of correctly aligned nucleotides in reads sampled from *Salmonella enterica subsp. enterica serovar Typhi str. CT18*. 100,000 Titanium reads averaging 521 bp were generated at an identity of 100% down to 95% using Flowsim [13]. The results were evaluated using FAAST with at *k *= 0 (Smith-Waterman-Gotoh algorithm) and *k *= 0.25 (FAAST algorithm), see Figure 3. As expected, both algorithms performed very well aligning close to all nucleotides correctly. However, FAAST were able to utilise the flowspace information to place more gaps correctly and thus gain a significant amount of correctly aligned nucleotides compared to the Smith-Waterman-Gotoh algorithm.

### Evaluating the performance of FAAST

As a straightforward performance test, FAAST was compared to NCBI BLAST 2 and SSAHA2 in a moderate sized alignment task. The test consisted of the typical task of aligning a set of sequenced reads against a small database. The query set was made up of Giardia P15 reads [14], produced using the Roche 454 GS FLX sequencing platform [2]. The sequencing run produced 221,245 reads, in total 45.8 Mbp, averaging 207 bp in length. The Giardia reads were queried against a database of all Giardia sequences in GenBank (2011-02-20), accessed through taxonomy identifier 5740. The database was composed of 11,178 sequences, in total 65.3 Mbp, see additional files for a complete list of files and scripts used in the evaluation.

The results of the alignment test are shown in table 1. FAAST finished the task in 6 minutes and 38 seconds, while SSAHA2 spent 4 min 48 sec and BLAST spent 28 min and 30 sec. SSAHA2 both spent the least wall time and spent the least CPU time showing outstanding efficiency. FAAST in comparison with BLAST was able to more efficiently make use of the multi-core CPU and BLAST actually exhausted slightly less CPU time. The Giardia sequencing run and the database can be downloaded through http://www.ifm.liu.se/bioinfo/.

**Table 1:** Performance evaluation of the FAAST program

Program	Wall time	CPU time	Memory
FAAST 1.0	6m38s	51m26s	348 Mb
SSAHA2 2.5.3	4m48s	4m48s	271 Mb
BLAST 2.2.21	28m30s	46m09s	37.8 Mb

Resources exhausted by FAAST, SSAHA2 and BLAST in alignment of 221,245 454 reads (45.8 Mbp in total) against a database of 11,178 sequences (65.3 Mbp in total). The test was performed on a 64-bit Linux machine with an 'Intel Core i7 920' processor, HT enabled. FAAST was executed with default parameters, thus indexing 11-mer nucleotides running in 8 threads and reporting 10 results per query. SSAHA2 was executed with the parameters '-kmer 11 -skip 2 -best 10 -seeds 2' and BLAST with '-v10 -b10 -a8' also indexing 11-mer nucleotides and reporting top 10 hits. The SSAHA2 and BLAST times do not include times for building the database (ssaha2Build and formatdb).

### FAAST - Flow-space Assisted Alignment Search Tool

In order to implement our algorithm, we have constructed an alignment search tool called FAAST (Flow-space Assisted Alignment Search Tool). FAAST is implemented as a C++ program and compatibility has been ensured using GNU GCC, Intel ICC 12.0 on Linux, but FAAST also compiles with minGW or Intel on the Windows platform. FAAST is an open source project and it is available both as pre-compiled Linux binaries and as source code at http://www.ifm.liu.se/bioinfo/. To facilitate searching with flow peak information, FAAST reads the SFF format (Standard Flowgram Format), which is used to pack 454 data. Furthermore, since the SFF format is a binary format that may be difficult to edit manually, a new format named FFASTA (Flowgram-FASTA) is supported. FFASTA is a FASTA-like format, but it expresses a flowgram for each entry instead of a nucleotide/amino acid sequence. In the FFASTA-format the flowgram is represented as an array of float values for each peak separated by white-space, just as the QUAL format for quality scores. FAAST is implemented with a wide range of parameters for adjusting indexing heuristics, the local alignment model, output-format etc. More information can be found in the FAAST documentation at http://www.ifm.liu.se/bioinfo/ (Under '454 Tools').

## Discussion

Due to the specific nature of pyrosequencing, mostly produced by 454 sequencing machines, regular Smith-Waterman-Gotoh alignments may be inadequate. A homopolymer reading error will introduce a gap in the alignment, which needs approximately 4-5 identities to be outweighed using typical alignment parameters. Thus, any homopolymer indel not flanked by a high enough number of identities will cause early termination of the alignment and/or erroneous alignments. By extending the model to allow the introduction of these gaps at a lower cost at points of homopolymer uncertainties, we show that alignment accuracy can be improved, see Figure 2. While flow-space assisted local alignment slightly decreases accuracy for non-pyrosequencing data, accuracy is gained for pyrosequencing data. Naturally, the improvement in alignment results also depends on the amount and lengths of homopolymer-tracts present in the original data as well as the complexity of the background, see Figure 2. We also note that with increasing sequence length the accuracy is higher at the same query identity level as 100 decoys sample less of the combinatorial space (i.e. there are more ways to place 4 SNPs in a 50 bp sequence then there are to place 2 SNPs in a 25 bp sequence). However, when then complexity of the background begin the affect the results the performance difference is similar regardless of nucleotide length. This is illustrated in Figure 3 where FAAST is able to improve the alignment results of full-length Titanium reads. However, since Titanium 454 data generally is of very high quality and trivially aligned, regular Smith-Waterman-Gotoh aligned over 99.5% of the nucleotides correctly and in total the gain of using FAAST could in some cases be considered small.

Even though FAAST was developed to deal with homopolymer reading errors of 454 data, the FAAST algorithm may be applied to other pyrosequencing methods or any sequencing method or data where homopolymer reading errors occur, for example the Ion Torrent Technology. Furthermore, many bioinformatic algorithms and software are based upon or use to some extent Smith-Waterman, for which the FAAST algorithm could be utilised to better handle homopolymer reading errors.

While BLAST relies on query indexing, FAAST uses database indexing, as implemented in the SSAHA alignment search tool [15]. This provides a speed advantage at the cost of requiring more RAM. The heuristics of FAAST is rudimentary and restricts the number of alignments performed simply through requiring *n *number of *k*-mer hits along the same diagonal not spaced more than *J *nucleotides apart. Although SSAHA2 is much faster that FAAST in a single CPU context, FAAST utilizes the multi-core environment well and still completes the alignment task evaluated in reasonable time. FAAST also compares fairly well to BLAST and in general it would be possible to use the FAAST software for producing alignments with 454 data.

## Conclusions

FAAST provides the possibility to both identify potential homopolymer reading errors in pyroseqencing data as well as providing more accurate alignments with pyrosequencing data. FAAST does not only provide high quality alignments but it does so using reasonable computational resources. Therefore, we propose that FAAST could serve as a useful tool in the analysis of genomic and metagenomic data as well as analysis where correctly aligned bases are vital, such as SNP detection.

## Methods

### FAAST alignment algorithm

The FAAST alignment algorithm is based on the Smith-Waterman-Gotoh algorithm. For a Smith-Waterman-Gotoh alignment using pyro-sequencing data, at least two maximum likelihood estimates are used. The first interprets the pyro-sequencing flowgram data into a nucleotide sequence and the second constitutes the actual alignment. By performing an alignment with flowpreak information, a single maximum likelihood estimate will be calculated, which eliminates error propagation from the first estimate to the second.

The local alignment can be solved using dynamic programming where each cell score, *D_i. j_*, is calculated through equation 1 (Smith-Waterman-Gotoh alignment).(1)

The variables, *d *and *q *represent the two sequences aligned where *d_i _*and *q_j _*are position *i *and *j *in the respective sequence. The function *s*(*d_i_*, *q_j_*) determines the score for aligning *d_i _*with *q_j_*. Finally, *E_i, j _*describes the optimal score of each cell that ends with a gap in the sequence *d *and *H_i, j _*describes the optimal score of each cell that ends with a gap in the sequence *q*. The two variables are calculated through *E_i, j_= *max(*E_i-1, j _- G_0_, E_i-1, j _- G_e_*) and *H_i, j _= *max(*H_i, j-1 _- G_0_, H_i, j-1 _- G_e_*) where *G_0 _*is the minimum gap penalty and *G_e _*is the gap extension penalty.

In the FAAST algorithm, calculations of each cell is substituted with *C_i, j _= *max(*D_i, j_, S_i, j_*). *D_i. j _*represents the Smith-Waterman-Gotoh score as defined in equation 1 and *S_i. j _*is the optimal score of each cell given a homopolymer correction in *(i, j)*, defined in equation 2.(2)

Here, *S_i. j _*is limited to describing a homopolymer correction up to four nucleotides. *Pd_j, n _*describes the 'down-calling' penalty for a query position *j*, where *n *is the number of bases by which the homopolymer is shortened. Accordingly, *Pu_j, n _*describes the 'up-calling' penalty for a query position *j *where *n *is the number of bases by which the homopolymer is lengthened. However, *Pu *is only evaluated as non-infinite when *d_i _*is equal to *q_j_*. Consequently, corrections are not allowed when a corresponding database nucleotide that can be corrected against is not found. For example, the query sequence "TAAT" would potentially align to "TAAAT" with a homopolymer correction in A, while it could not be aligned against "TAACT".

**Figure 4:**
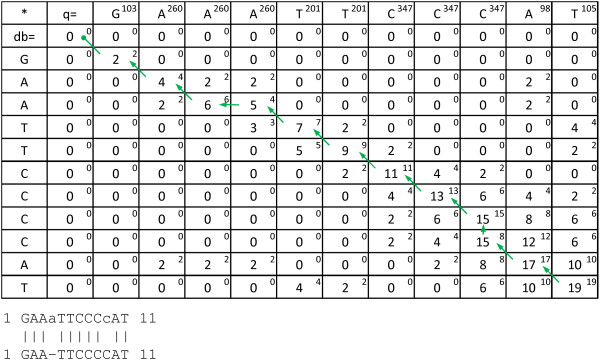
**An example of a FAAST alignment dynamics programming matrix**. Showing the calculated FAAST alignment where one peak (260, A) has been identified as a potential overcall (penalized with -1) and another peak (347, C) have been identified as a likely undercall (not penalized). In this example a nucleotide match score, M = [2, -3] and a gap penalty of G = [5,2] were used. The score of each cell *C_i, j _*is marked in the middle and the non-homopolymer score *D_i, j _*is marked in each upper right corner. Each flowpeak value is also indicated in each upper right corner of the query sequence (first row). Homopolymer corrections are marked in the produced alignment by lower-case characters, either inserted as with undercalls or aligned towards a database sequence gap as with overcalls. The corresponding Smith-Waterman-Gotoh alignment would result in an alignment of the non-gaped middle part of both queries ("AATTCCC").

Both vectors *Pd *and *Pu *can be pre-calculated for a query sequence and the penalties are only set to non-infinite if *j *is the last position of a homopolymer and the penalties are smaller than the penalty for a corresponding normal gap. Since a longer homopolymer gap is more (or for extreamly long homopolymers equally) penalised than a shorter (*Pd_i, j, n _*≤ *Pd_i, j, n+1_*), if *Pd_i, j, n _*is infinite, so is *Pd_i, j, n+1 _*and it does not need to be included in the calculation of *S_i, j_*. An example of a FAAST alignment is shown in Figure 4.

### Homopolymer correction penalties

Given a flowpeak value, *f*, the peak will be called as an *n*-homopolymer, where *n *is the rounded integer value of the flowpeak value. The deviation in flowpeak value required to call the peak as a *m*-homopolymer can be calculated given equation 3.(3)

**Figure 5:**
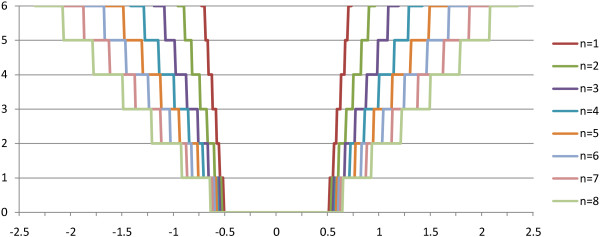
**Homopolymer deviation penalties**. Illustrating homopolymer correction penalties as calculated in FAAST, for peak-value deviations ranging from -2.5 to 2.5 for homopolymers of length *n *= [1,8]. The maximum allowed flowpeak range, *k = *0.25 (25%), as well as the minimum gap penalty of 7 (corresponds to the proportionality constant *α *= 28) was used for the calculation were the calculated penalty is rounded to the closest integer.

Homopolymer correction penalties are in FAAST proportional to this minimum flowpeak deviation, thus calculated through P = α *Dev_m_/n, where *α *is the proportionality constant. The homopolymer correction penalty, *P*, could alternatively be described relative to the minimum gap penalty *G_0 _*through P = G_0 _*(*Dev_m_/n*)*/k *and thus *k *describes at which relative flowpeak deviation, *Dev_m_/n*, one would obtain a homopolymer penalty equal to the minimum gap penalty, thus also the maximum flowpeak correction allowed. The gain of specifying penalties through *k *is a gap-penalty agnostic parameter as well as a parameter that is easier for the users to understand. Notably, the use of *k = 0 *would revert the FAAST algorithm into Smith-Waterman-Gotoh as homopolymer penalties would be infinitely large and thus *C_i, j _= D_i, j_*. An example of homopolymer penalties in FAAST at various flowpeak value deviations for different *n*-mers is shown in Figure 5. As can be seen the homopolymer correction penalty, *P*, is in theory proportional to *Dev_m _*and inversely proportional to *k*, while integer rounding and non-centred (non-integer) flow-peak values often make it non-linear.

### Indexing and heuristics

The first part of the search algorithm is composed of constructing a database index, by which the occurrence of any *k*-tuple can be requested. FAAST employs a direct and compact database indexing model where the occurrences of all valid *k*-tuples and the corresponding database position are noted, in the same way as in SSAHA [15]. All occurrences (database positions) are noted in a list, *L*. Each *k*-tuple of valid nucleotides ("T", "A", "C" or "G") is then treated as an integer value of base 4 with the 4 nucleotides as alphabet. A second pointer list, *P*, of size 4*^k ^*is allocated and populated so that *P *holds a pointer to *L *for each *k*-tuple of valid nucleotides. Finally, *L *and *P *are ordered so that each position of the list *P *points to the first occurrence of the corresponding *k*-tuple in *L *and the value of *P_i _*<*P_i+1_*. Consequently, the last occurrence of any *k*-tuple found at position, *i*, is retrieved through reading the first occurrence of the next *k*-tuple found in position, *i *+ 1, in *P*.

Through matching all *k*-tuples found in each query against the database-index, all positions at which the query and database share at least *k *nucleotides are found, denoted as a 'hit'. The 'hits' are subsequently sorted by diagonal and an alignment is generated if at least *n *hits are found on the same diagonal spaced less than *J *nucleotides apart. Finally, for the *v *top-scoring alignments, a re-alignment with complete trace is performed to enable full-alignment output. The default parameters of FAAST use *k *= 11, *n *= 2 and *J *= 50, thus requiring at least two 11-mer 'hits' spaced no more than 50 bp apart.

## Competing interests

The authors declare that they have no competing interests.

## Authors' contributions

FL has designed the algorithm and implemented the FAAST software and written the manuscript. BP and BA have helped to design the study and draft the manuscript and have provided feedback on the algorithm. All authors read and approved the final manuscript.

## Supplementary Material

Additional file 1 **Evaluation of the effect of flow-space assisted local alignment**. Scripts for running the evaluation test as well as all the binaries and results from the run included in the manuscript. See the enclosed README.txt for more information. Click here for file

Additional file 2 **Evaluating the performance of FAAST**. Scripts for running the evaluation test as well as all the binaries and results from the run included in the manuscript. See the enclosed README.txt for more information. Click here for file
